# Immunogenicity and Pre-Clinical Efficacy of an OMV-Based SARS-CoV-2 Vaccine

**DOI:** 10.3390/vaccines11101546

**Published:** 2023-09-29

**Authors:** Alberto Grandi, Michele Tomasi, Irfan Ullah, Cinzia Bertelli, Teresa Vanzo, Silvia Accordini, Assunta Gagliardi, Ilaria Zanella, Mattia Benedet, Riccardo Corbellari, Gabriele Di Lascio, Silvia Tamburini, Elena Caproni, Lorenzo Croia, Micol Ravà, Valeria Fumagalli, Pietro Di Lucia, Davide Marotta, Eleonora Sala, Matteo Iannacone, Priti Kumar, Walther Mothes, Pradeep D. Uchil, Peter Cherepanov, Martino Bolognesi, Massimo Pizzato, Guido Grandi

**Affiliations:** 1 Toscana Life Sciences Foundation, Via Fiorentina 1, 53100 Siena, Italy; a.grandi@toscanalifesciences.org (A.G.); a.gagliardi@toscanalifesciences.org (A.G.); m.benedet@toscanalifesciences.org (M.B.); g.dilascio@toscanalifesciences.org (G.D.L.); e.caproni@toscanalifesciences.org (E.C.); 2 BiOMViS Srl, Via Fiorentina 1, 53100 Siena, Italy; 3 Department of Cellular, Computational and Integrative Biology (CIBIO), University of Trento, Via Sommarive 9, 38123 Trento, Italy; michele.tomasi.2@unitn.it (M.T.); cinzia.bertelli@unitn.it (C.B.); teresa.vanzo@unitn.it (T.V.); silvia.accordini@unitn.it (S.A.); ilaria.zanella@unitn.it (I.Z.); riccardo.corbellari@unitn.it (R.C.); silvia.tamburini@unitn.it (S.T.); lorenzo.croia@unitn.it (L.C.); 4 Section of Infectious Diseases, Department of Internal Medicine, School of Medicine, Yale University, New Haven, CT 06520, USA; irfan.ullah@yale.edu (I.U.); walther.mothes@yale.edu (W.M.); pradeep.uchil@yale.edu (P.D.U.); 5 Division of Immunology, Transplantation and Infectious Diseases, IRCCS San Raffaele Scientific Institute, 20132 Milan, Italy; rava.micol@hsr.it (M.R.); fumagalli.valeria@hsr.it (V.F.); dilucia.pietro@hsr.it (P.D.L.); marotta.davide@hsr.it (D.M.); sala.eleonora@hsr.it (E.S.); iannacone.matteo@hsr.it (M.I.); 6 Vita-Salute San Raffaele University, Via Olgettina 58, 00132 Milan, Italy; 7 Experimental Imaging Center, IRCCS San Raffaele Scientific Institute, 20132 Milan, Italy; 8 Department of Microbial Pathogenesis, School of Medicine, Yale University, New Haven, CT 06510, USA; priti.kumar@yale.edu; 9 Chromatin Structure and Mobile DNA Laboratory, The Francis Crick Institute, London NW1 1AT, UK; peter.cherepanov@crick.ac.uk; 10 Biosciences Department, University of Milan, Via Celoria 26, 20133 Milan, Italy; martino.bolognesi@unimi.it

**Keywords:** SARS-CoV-2, OMV-based vaccine, RBD neutralizing epitopes, OMV engineering, cross-protective vaccine

## Abstract

The vaccination campaign against SARS-CoV-2 relies on the world-wide availability of effective vaccines, with a potential need of 20 billion vaccine doses to fully vaccinate the world population. To reach this goal, the manufacturing and logistic processes should be affordable to all countries, irrespective of economical and climatic conditions. Outer membrane vesicles (OMVs) are bacterial-derived vesicles that can be engineered to incorporate heterologous antigens. Given the inherent adjuvanticity, such modified OMVs can be used as vaccines to induce potent immune responses against the associated proteins. Here, we show that OMVs engineered to incorporate peptides derived from the receptor binding motif (RBM) of the spike protein from SARS-CoV-2 elicit an effective immune response in vaccinated mice, resulting in the production of neutralizing antibodies (nAbs) with a titre higher than 1:300. The immunity induced by the vaccine is sufficient to protect the animals from intranasal challenge with SARS-CoV-2, preventing both virus replication in the lungs and the pathology associated with virus infection. Furthermore, we show that OMVs can be effectively decorated with the RBM of the Omicron BA.1 variant and that such engineered OMVs induce nAbs against Omicron BA.1 and BA.5, as measured using the pseudovirus neutralization infectivity assay. Importantly, we show that the RBM_438–509_ ancestral-OMVs elicited antibodies which efficiently neutralize in vitro both the homologous ancestral strain, the Omicron BA.1 and BA.5 variants with a neutralization titre ranging from 1:100 to 1:1500, suggesting its potential use as a vaccine targeting diverse SARS-CoV-2 variants. Altogether, given the convenience associated with the ease of engineering, production and distribution, our results demonstrate that OMV-based SARS-CoV-2 vaccines can be a crucial addition to the vaccines currently available.

## 1. Introduction

The dramatic SARS-CoV-2 pandemic exploded worldwide at the beginning of 2020 and has triggered an unprecedented race to the development of effective vaccines. In less than a three-year timeframe, hundreds of vaccines have been designed and tested in preclinical settings, more than 100 have reached the clinic, and 24 are currently authorized for human use [1]. It is estimated that more than 9 billion doses have been administered so far worldwide, saving approximately 1 million lives.

Despite this spectacular success of modern vaccinology, the race against COVID-19 is not over. Mostly because of costs and logistic issues, vaccine distribution is heavily unbalanced, with half of the planet still waiting for a dose and with only 4% of populations in low-income countries being vaccinated [2]. Moreover, SARS-CoV-2 has the extraordinary capacity to continuously accumulate mutations, which allow the virus to escape, at least partially, host immune responses while preserving infectivity and virulence [3].

To overcome such challenges and to provide a sustainable long-term prophylaxis, a “pan-vaccine” capable of eliciting a broad, cross-protective immune response should become available. The ideal vaccine would negate the need of booster immunizations using vaccines tailored for the emerging variants of concern (VOCs). In addition, the vaccine should rely on easily scalable, low-cost production processes, while not requiring the cold chain to simplify world-wide distribution.

Among the several technologies available for vaccine development, outer membrane vesicles (OMVs) have emerged in recent years as an attractive tool capable of coupling excellent built-in adjuvanticity provided by the microbe-associated-molecular patterns (MAMPs) embedded in the vesicles, and an easily scalable production and purification process [4]. Anti-Neisseria OMV-based vaccines are currently available for human use [5], and others against Shigella and Salmonella are in advanced clinical phases [6,7].

We have recently developed a platform based on “proteome minimized” *E. coli* OMVs selectively loaded with heterologous antigens [8]. The platform has been successfully applied to design prophylactic vaccines against infectious diseases [9] and was shown to stimulate potent anti-tumour activity in different mouse models [10,11].

Because OMVs are readily phagocytosed, the associated antigens are efficiently presented by professional antigen-presenting cells, eliciting both antibody- and T-cell responses [12]. The reaction is coupled to the production of IFN-γ, ensuring a sustained Th1 response as well as an optimal humoral response. Clinical evidence demonstrates that an accelerated induction of a Th1 cell response is associated with less severe cases of COVID-19 [13,14]. Moreover, convalescent individuals tend to develop strong memory CD4+ and CD8+ T cells [15]. Therefore, the ability of OMVs to trigger Th1 represents a desired feature. Crucially, in addition to the simplicity and cost-effectiveness of OMV production pipelines [16], the antigen-decorated vesicles are extremely stable for long-term storage at room temperature, potentially making them ideal vaccine candidates for world-wide distribution.

Essentially, all available vaccines and those under development are designed to induce antibodies specific for the spike (S) protein or its receptor binding domain (RBD). Neutralizing titres found in vaccinees or in convalescent individuals correlate strongly with antibody binding to the RBD [17]. Moreover, the most potent monoclonal antibodies isolated from convalescent patients recognize epitopes located within the receptor binding motif (RBM), the RBD region which directly engages the viral receptor, angiotensin-converting enzyme 2 (ACE2) [18]. The ability to specifically concentrate the immune response against these epitopes would exclusively elicit neutralizing antibodies, while minimizing the generation of non-neutralizing or poorly neutralizing immunoglobulins binding to irrelevant spike regions.

The OMVs offer the unique opportunity to display short and defined epitopes to B- and T-cells in a highly immunogenic context provided by the bacterial vesicle. Taking advantage of such potential and the availability of the crystal structure of the RBD in complex with ACE2, we engineered the OMVs with peptides from the SARS-CoV-2 ancestral RBM. Here, we show that RBM-derived peptides can be expressed in the OMV membrane and induce neutralizing antibodies sufficient to fully protect human ACE2 (hACE2)-transgenic mice from a challenge with SARS-CoV-2. We also show that the same region from the BA.1 variant can also be efficiently expressed in the OMVs. Moreover, the RBM_438–509_ ancestral-OMVs elicit antibodies which can neutralize in vitro both the homologous ancestral strain and the Omicron BA.1 and the BA.5 variants, indicating that OMV-RBM formulations elicit novel populations of antibodies with robust cross-reactive neutralization capacity.

Given the efficacy of the vaccine in the animal model, the cross-neutralization capacity, the ease of its engineering and the cost-effective production process, we propose the RBM_438–509_ ancestral-OMVs as a promising candidate to continue the vaccination campaign against SARS-CoV-2.

## 2. Materials and Methods

### 2.1. Engineering BL21(DE3) E. coli Strains with SARS-CoV-2 Neutralizing Epitopes

The pET21-FhuD2 plasmid carrying the *Staphylococcus aureus* Ferric hydroxamate receptor 2 (FhuD2) was fused to one copy of RBM_438–462,_ RBM_467–509_ and RBM_438–509_ SARS-CoV-2 epitope, respectively. The three plasmids were assembled using the PIPE method [19]. Briefly, pET21-FhuD2 was linearized by PCR, using FhuD2-v-R and pET-V-F primers (Table 1). In parallel, the synthetic DNA encoding the RBM of SARS-CoV-2 was synthesized by GeneArt (Thermo Fisher Scientific, Waltham, MA, USA) and used as a template for the amplification of the three epitopes. In detail, RBM_438–462_ and RBM_467–509_ (Table 2) were amplified by PCR with the forward 2-F and 1-F and the reverse 2-R and 1-R primers, respectively. The PCR products and the linearized plasmid were mixed together and used to transform the *E. coli* HK100 strain.

The RBM_438–509_, the combination of the two epitopes, was assembled with two steps of PCR in succession. In the first step, two different fragments carrying an overlapping sequence were amplified with 2-F/R-1 and F-1/1-R primers. In the second step, the two fragments were eluted from Agarose gel, and mixed and used as template for a final PCR reaction with the primers 2-F and 1-R. This final product and the linearized plasmid were mixed and used to transform the *E. coli* HK100 strain.

The RBD-BA.1_438–509_ gene was synthesized by GeneArt (Thermo Fisher Scientific, Waltham, MA, USA) and used as template for the amplification of the RBM-BA.1_438–509_ using the CoSASta-F and CoSASta-R primers. The low copy number pACYC plasmid was linearized using the couple of primers FhuD2-v-R and PACYC-F The PCR product and the linearized plasmid were mixed and used to transform *E. coli* HK100 strain.

To confirm the correct gene fusions, plasmids were sequenced (Eurofins, Ebersberg, Germany) and the *E. coli* BL21(DE3)*Δ60* strain was transformed with pET21-FhuD2-RBM_438–462,_ pET21-FhuD2-RBM_467–509_, pET21-FhuD2-RBM_438–509_ and pACYC-FhuD2-RBM-BA.1_438–509_ plasmids and the derived recombinant strains were used for the production of engineered FhuD2-RBM_438–462,_ FhuD2-RBM_467–509,_ FhuD2-RBM_438–509_ (RBM_ancestral-_OMVs) and FhuD2-RBM-BA.1_438–509_ (RBM_BA_._1-_OMVs) OMVs, respectively.

### 2.2. OMV Purification

*OMVs* from K-12 *E. coli* BL21(DE3)*Δ60*-pET-FhuD2-RBM_438–509_, BL21(DE3)*Δ60*-pET-FhuD2-RBM_438–462_, BL21(DE3)*Δ60*-pET-FhuD2-RBM_467–509_ and BL21(DE3)*Δ60-*pACYC-FhuD2-RBMBA.1_438–509_ were purified in an EZ control bioreactor (Applikon Biotechnology, Schiedam, The Netherlands) as previously described [8]. Cultures were started at an OD_600_ of 0.1 and grown in LB medium at 30 °C, pH 6.8 (±0.2), dO_2_ > 30%, 280–500 rpm until OD_600_ = 0.5, then the temperature was lowered to 25 °C. The expression of the recombinant protein was induced when the culture reached an OD_600_ of 1 with 0.1 mM IPTG and a feed made of 50 mg/L ampicillin, 15 g/L glycerol, 0.25 g/L MgSO_4_ was added to the culture medium. The fermentation was carried out until the end of the exponential phase at 25 °C. OMVs were then purified and quantified as previously described [8]. Culture supernatants were separated from biomass by centrifugation at 4000× *g* for 20 min. After filtration through a 0.22-μm pore size filter (Millipore, Burlington, MA, USA), OMVs were isolated, concentrated and diafiltrated from the supernatants using Tangential Flow Filtration (TFF) with a Cytiva Äkta Flux system. OMVs were quantified using a DC protein assay (Bio-Rad, Hercules, CA, USA) and their protein content analysed by loading 20 μg of total protein on SDS-PAGE.

### 2.3. Studies with CD1 Mice

Mice were monitored twice per day to evaluate early signs of pain and distress, such as respiration rate, posture, and loss of weight (more than 20%) according to humane endpoints. Animals showing such conditions were anesthetized and subsequently sacrificed in accordance with experimental protocols, which were reviewed and approved by the Animal Ethical Committee of The University of Trento and the Italian Ministry of Health.

Five-week-old CD1 female mice were immunized intraperitoneally (i.p.) on day 0, 14 and 28 with 10 μg of OMVs together with 2 mg/mL Aluminium hydroxide in a final volume of 200 µL. Sera were collected 7 days after the last immunization. Alternatively, mice received intramuscularly (i.m.) 50 µL of AstraZeneca ChAdOx1 nCoV-19 vaccine (AZ), consisting of roughly 2.5 × 10^7^ infectious units.

### 2.4. Studies with the ACE2 Mouse Model

B6.Cg-Tg(K18-hACE2)^2Prlmn/^J mice [1] were purchased from The Jackson Laboratory. Mice were housed under specific pathogen-free conditions and heterozygous mice were used at 8–10 weeks of age. All experimental animal procedures were approved by the Institutional Animal Committee of the San Raffaele Scientific Institute and all infectious work was performed in designed BSL-3 workspaces. The hCoV-19/Italy/LOM-UniSR-1/2020 (EPI_ISL_413489) isolate of SARS-CoV-2 was obtained from the Unit of Microbiology and Virology of San Raffaele Scientific Institute and grown in Vero E6 cells. K18-hACE2-transgenic mice were i.p. immunized twice, 14 days apart, with 10 µg vaccine in 200 μL of PBS or with PBS alone. Virus infection was performed via intranasal administration of 1 × 10^5^ TCID_50_ SARS-CoV-2 per mouse. Tissue homogenates were prepared by homogenizing perfused lung using gentleMACS Octo Dissociator (Miltenyi Biotec, #130-096-427, Bergisch Gladbach, Germany) in M tubes (#130-093-335) containing 1 mL of DMEM. Samples were homogenized three times with the program m_Lung_01_02 (34 s, 164 rpm). The homogenates were centrifuged at 3.500 rpm for 5 min at 4 °C. The supernatant was collected and stored at −80 °C for viral isolation and viral load detection. Viral titres were calculated by 50% tissue culture infectious dose (TCID_50_). Briefly, Vero E6 cells were seeded at a density of 1.5 × 10^4^ cells per well in flat-bottom 96-well tissue-culture plates. The following day, 2-fold dilutions of the homogenized tissue were applied to confluent cells and incubated for 1 h at 37 °C. Then, cells were washed with phosphate-buffered saline (PBS) and incubated for 72 h at 37 °C in DMEM 2% FBS. Cells were fixed with 4% paraformaldehyde for 20 min and stained with 0.05% (*w*/*v*) crystal violet in 20% methanol.

### 2.5. RNA Extraction and qPCR

Tissue homogenates were prepared by homogenizing perfused lungs using gentleMACS dissociator (Miltenyi Biotec, #130-096-427, Bergisch Gladbach, Germany ) with program RNA_02 in M tubes (#130-096-335) in 1 mL of Trizol (Invitrogen, #15596018, Waltham, MA, USA). The homogenates were centrifuged at 2000× *g* for 1 min at 4 °C and the supernatant was collected. RNA extraction was performed by combining phenol/guanidine-based lyisis with silica membrane-based purification. Briefly, 100 µL of Chloroform was added in 500 mL of homogenized sample and the total RNA was extracted using a ReliaPrep™ RNA Tissue Miniprep column (Promega, Cat #Z6111, Madison, WI, USA). Total RNA was isolated according to the manufacturer’s instructions. qPCR was performed using TaqMan Fast virus 1 Step PCR Master Mix (Life Technologies #4444434, Carlsbad, CA, USA), the standard curve was drawn with 2019_nCOV_N Positive control (IDT#10006625), and the primers used are 2019-nCoV_N1-Forward Primer (5′-GAC CCC AAA ATC AGC GAA AT-3′), 2019-nCoV_N1-Reverse Primer (5′-TCT GGT TAC TGC CAG TTG AAT CTG-3′) 2019-nCoV_N1-Probe (5′-FAM-ACC CCG CAT TAC GTT TGG TGG ACC-BHQ1-3′) (Centers for Disease Control and Prevention (CDC), Atlanta, GA, USA). All experiments were performed in duplicate.

### 2.6. Cell Isolation and Flow Cytometry

Mice were euthanized by cervical dislocation. Lungs were perfused through the right ventricle with PBS at the time of autopsy. Lung tissue was digested in RPMI 1640 containing 3.2 mg/mL Collagenase IV (Sigma, #C5138, St. Louis, MO, USA) and 25 U/mL DNAse I (Sigma-Aldrich, #D4263, St. Louis, MO, USA) for 30 min at 37 °C. Homogenized lungs were passed through a 70 μm nylon mesh to obtain a single-cell suspension. Cells were resuspended in 36% percoll solution (Sigma #P4937, St. Louis, MO, USA) and centrifuged for 20 min at 2000 rpm (light acceleration and low brake). The remaining red blood cells were removed with ACK lysis. Cell viability was assessed by staining with Viobility™ 405/520 fixable dye (Miltenyi Biotec, Cat #130-109-814, Bergisch Gladbach, Germany). The antibodies (Abs) used are presented in Table S1. Flow cytometry analysis was performed on a BD FACSymphony A5 SORP and analysed with FlowJo software V10 6.1 (Treestar).

### 2.7. Confocal Immunofluorescence Histology and Histochemistry

Lungs of infected mice were collected and fixed in 4% paraformaldehyde (PFA). Samples were then dehydrated in 30% sucrose prior to embedding in OCT freezing media (Bio-Optica, Milan, Italy). Twenty micrometre sections were cut on a CM1520 cryostat (Leica, Wetzlar, Germany) and adhered to Superfrost Plus slides (Thermo Fisher Scientific, Waltham, MA, USA). Sections were then permeabilized and blocked in PBS containing 0.3% Triton X-100 (Sigma-Aldrich, St. Louis, MO, USA) and 5% FBS followed by staining in PBS containing 0.3% Triton X-100 and 1% FBS. Slides were stained for SARS-CoV-2 nucleocapsid (GeneTex, Irvine, CA, USA) for 1 h RT. Then, slides were stained with Alexa Fluor 568 Goat Anti-Rabbit antibody for 2 h RT. All slides were analysed by confocal fluorescence microscopy (Leica TCS SP5 Laser Scanning Confocal). For N-SARS-CoV-2 immunohistochemistry, mice were perfused with PBS and lungs were collected in Zn-formalin and transferred into 70% ethanol 24 h later. Tissue was then processed, embedded in paraffin and automatically stained for SARS-CoV-2 (2019-nCoV) Nucleocapsid Antibody (SINO BIO, 40143-R019, Beijing, China) through LEICA BOND RX 1 h RT and developed with Bond Polymer Refine Detection (Leica Biosystem, DS9800, Mannheim, Germany). Brightfield images were acquired through an Aperio Scanscope System CS2 microscope and an ImageScope program (Leica Biosystem, Mannheim, Germany) following the manufacturer’s instructions. In both immunofluorescence and histochemistry, the N-SARS-CoV-2 percentage of positive area was determined by QuPath V0.4.4 (Quantitative Pathology & Bioimage Analysis) software.

### 2.8. Clinical Score

Mice were observed daily for clinical symptoms. Disease severity was scored as reported in Table 3.

### 2.9. Studies with hACE2-Transgenic B6 Mice Using Bioluminescent Reporter Viruses

All animals were maintained in the (SPF-free) barrier facility of the Yale University Animal Resource Centre (YARC) in a 14:10 light/dark cycle. All SARS-CoV-2-infected animals were housed in an animal room under BSL3 containment. The cages, animal waste, bedding, and carcasses of animals were disposed of and decontaminated in accordance with the guidelines set by Environmental Health Services at Yale. The replication-competent virus-infected animals were handled under ABSL3 conditions. All experiments described here were approved by Institutional Animal Care and Use Committee (IACUC) as well as SOPs approved by the Institutional Environmental Health and Biosafety committee. hACE2-transgenic B6 mice (heterozygous) were obtained from Jackson Laboratory. Six-to-eight-week-old male and female mice were used for all the experiments. The heterozygous mice were crossed and genotyped to select heterozygous mice for experiments by using the primer sets recommended by Jackson Laboratory. Each cohort size was n = 5 to allow for statistical testing. We calculated the number of animals (n = 4–8 per cohort) needed to achieve statistically significant results using an a priori power analysis. We calculated power and sample sizes required based on data from pilot experiments and previous studies [20,21,22]. Animals with sex- and age-matched littermates were included randomly in the experiments. Animals were not excluded due to illness after the experiments. To ensure that the sex of the animals does not constitute a biological variable during analysis, equal numbers of male and female mice were included whenever possible.

### 2.10. Cell and Viruses

Vero E6 (CRL-1586, American Type Culture Collection (ATCC), were cultured at 37 °C in RPMI supplemented with 10% foetal bovine serum (FBS), 10 mM HEPES pH 7.3, 1 mM sodium pyruvate, 1× non-essential amino acids, and 100 U/mL of penicillin–streptomycin. SARS-CoV-2/USA_WA1/2019 isolate expressing nanoluc luciferase (nLuc) was obtained from Craig B Wilen, Yale University and generously provided by K. Plante and Pei-Yong Shi, World Reference Center for Emerging Viruses and Arboviruses, University of Texas Medical Branch [23]. Viruses were propagated in Vero E6 TMPRSS2 by infecting them in T150 cm^2^ flasks at an MOI of 0.1. The culture supernatants were collected after 18 h when cytopathic effects were clearly visible. The cell debris was removed by sedimentation and filtered through 0.45 micron filter to generate virus stocks. Viruses were concentrated by adding one volume of cold (4 °C) 4× PEG-it Virus Precipitation Solution (40% (*w*/*v*) PEG-8000 and 1.2 M NaCl; System Biosciences) to three volumes of virus-containing supernatant. The solution was mixed by inverting the tubes several times and then incubated at 4 °C overnight. The precipitated virus was harvested by centrifugation at 1500× *g* for 60 min at 4 °C. The concentrated virus was then resuspended in PBS then aliquoted for storage at −80 °C. All work with infectious SARS-CoV-2 reporter viruses was performed in Institutional Biosafety Committee of Yale University (IBCYU)-approved BSL3 and A-BSL3 facilities at Yale University School of Medicine using appropriate positive pressure air respirators and protective equipment.

### 2.11. OMV Vaccination and SARS-CoV-2 Infection

For all bioluminescence imaging-based in vivo experiments, groups of five 6-to-8-week-old K18 hACE-transgenic mice (both male and female) were immunized with 10 µg of either “empty OMVs” or RBM_ancestral-_OMVs both intramuscularly (i.m., 50 µL per mouse; mixed with 2 mg/mL aluminium hydroxide (Alum) on the day of immunization, day 0) and intranasally (i.n., 25 µL, without any adjuvant). The animals were then boosted on day 14 and day 28 both i.m and i.n in a similar manner as primary vaccination. On day 35, the mice were intranasally challenged with 1 × 10^5^ FFU SARS-CoV-2_WA1_nLuc in 25–30 µL volume under anaesthesia (0.5–5% isoflurane delivered using precision Dräger vaporizer with an oxygen flow rate of 1 L/min). The starting body weight was set to 100%. For survival experiments, mice were monitored every 8–12 h starting six days after virus challenge. Lethargic and moribund mice or mice that had lost more than 20% of their body weight were sacrificed and considered to have succumbed to infection for Kaplan–Meier survival plots. Mice were considered to have recovered if they gained back all the lost weight (experimental endpoint).

### 2.12. Bioluminescence Imaging (BLI) of SARS-CoV-2 Infection

All standard operating procedures and protocols for IVIS imaging of SARS-CoV-2-infected animals under ABSL-3 conditions were approved by IACUC, IBSCYU and Yale Animal Research Center (YARC). All the imaging was carried out using IVIS Spectrum^®^ (Perkin Elmer, Waltham, MA, USA) in XIC-3 animal isolation chamber (Perkin Elmer, Waltham, MA, USA) that provided biological isolation of anesthetized mice or individual organs during the imaging procedure. All K18 hACE-transgenic mice were anesthetized via isoflurane inhalation (3–5% isoflurane, oxygen flow rate of 1.5 L/min) prior and during BLI using the XGI-8 Gas Anesthesia System. Prior to imaging, 100 µL of nanoluc luciferase (nLuc) substrate, furimazine (NanoGlo^TM^, Promega, Madison, WI, USA) diluted 1:40 in endotoxin-free PBS was retro-orbitally administered to mice under anaesthesia. The mice were placed into XIC-3 animal isolation chamber (Perkin Elmer, Waltham, MA, USA) pre-saturated with isothesia and oxygen mix. The mice were imaged in both dorsal and ventral position at indicated days post infection. The animals were then imaged again after euthanasia and necropsy by spreading an additional 200 µL of substrate onto the exposed intact organs. Infected areas identified by carrying out whole-body imaging after necropsy were isolated, washed in PBS to remove residual blood and placed onto a clear plastic plate. Additional droplets of furimazine in PBS (1:40) were added to organs and soaked in substrate for 1–2 min before BLI. Images were acquired and analysed with Living Image v4.7.3 in vivo software package (Perkin Elmer, Waltham, MA, USA). Image acquisition exposures were set to auto, with imaging parameter preferences set in order of exposure time, binning, and f/stop, respectively. Images were acquired with luminescent f/stop of 2, photographic f/stop of 8. Binning was set to medium. Comparative images were compiled and batch-processed using the image browser with collective luminescent scales. Photon flux was measured as luminescent radiance (p/sec/cm^2^/sr). During luminescent threshold selection for image display, luminescent signals were regarded as background when minimum threshold setting resulted in displayed radiance above non-tissue-containing or known uninfected regions.

### 2.13. Focus Forming Assay

Titres of virus stocks were determined using a standard plaque assay. Briefly, the 4 × 10^5^ Vero-E6 cells were seeded on a 12-well plate. Then, 24 h later, the cells were infected with 200 µL of serially diluted virus stock. After 1 h, the cells were overlayed with 1 mL of pre-warmed 0.6% Avicel (RC-581 FMC BioPolymer, Philadelphia, PA, USA) made in complete RPMI medium. Plaques were resolved at 48 h post infection by fixing in 10% paraformaldehyde for 15 min followed by staining for 20 min with 0.2% crystal violet made in 20% ethanol. Plates were rinsed in water to visualize plaques.

### 2.14. Measurement of Viral Burden

Indicated organs (nasal cavity, brain, and lungs) from infected or uninfected mice were collected, weighed, and homogenized in 1 mL of serum-free RPMI media containing penicillin–streptomycin and homogenized in a 2 mL tube containing 1.5 mm Zirconium beads with BeadBug 6 homogenizer (Benchmark Scientific, TEquipment Inc., Long Branch, NY, USA). Virus titres were measured using three highly correlative methods. First, the total RNA was extracted from homogenized tissues using an RNeasy plus Mini kit (Qiagen Cat # 74136, Hilden, Germany), reverse-transcribed with an iScript advanced cDNA kit (Bio-Rad Cat #1725036, Hercules, CA, USA) followed by a SYBR Green Real-time PCR assay for determining copies of SARS-CoV-2 N gene RNA using primers SARS-CoV-2 N F: 5′-ATGCTGCAATCGTGCTACAA-3′ and SARS-CoV-2 N R: 5′-GACTGCCGCCTCTGCTC-3′. All our real-time PCR assays based on SYBR Green had a built-in melt-curve that was checked to ensure estimation of only specific PCR products and not false positives. Second, we used Nanoluc (nLuc) luciferase activity as a shorter surrogate for plaque assay. Infected cells were washed with PBS and then lysed using 1× Passive lysis buffer. The lysates transferred into a 96-well solid white plate (Costar Inc., Washington, DC, USA) and nLuc activity was measured using a Tristar multiwell Luminometer (Berthold Technology, Bad Wildbad, Germany) for 2.5 s by adding 20 µL of Nano-Glo^®^ substrate in a Nanoluc assay buffer (Promega Inc., Madison, WI, USA). Normalized relative light units were determined using uninfected monolayers of Vero cells treated identically. The data were processed and plotted using GraphPad Prism 8 v8.4.3.

### 2.15. Analyses of Signature Inflammatory Cytokines mRNA Expression

Brain and lung samples were collected from mice at the time of necropsy. Approximately 20 mg of tissue was suspended in 500 µL of RLT lysis buffer, and RNA was extracted using an RNeasy plus Mini kit (Qiagen Cat # 74136), reverse-transcribed with an iScript advanced cDNA kit (Bio-Rad Cat #1725036). To determine mRNA copy numbers of signature inflammatory cytokines, multiplex qPCR was conducted using iQ Multiplex Powermix (BioRad Cat # 1725848, Hercules, CA, USA) and PrimePCR Probe Assay mouse primers FAM-GAPDH, HEX-IL6, TEX615-CCL2, Cy5-CXCL10, and Cy5.5-IFNgamma. All PrimePCR Probe Assay mouse primers were purchased from Bio-Rad. The reaction plate was analysed using CFX96 touch real time PCR detection system. All channels were set to scan mode. The PCR conditions were 95 °C 2 min, 40 cycles of 95 °C for 10 s and 60 °C for 45 s, followed by a melting curve analysis to ensure that each primer pair resulted in amplification of a single PCR product. mRNA copy numbers of *Il6*, *Ccl2*, *Cxcl10* and *Ifng* in the cDNA samples of infected mice were normalized to *Gapdh* mRNA with the formula ΔC_t_(target gene) = C_t_(target gene) − C_t_(*Gapdh*). The fold increase was determined using the 2^−ΔΔCt^ method comparing treated mice to uninfected controls.

### 2.16. ELISA

Ninety-six-well Maxisorp plates (Nunc, Thermo Fisher Scientific) were coated with 200 ng/well of purified recombinant RBD and blocked with 100 mL/well of PBS + 1% BSA. Mice sera were threefold serially diluted in PBS + 1% BSA starting from a 1:100 initial dilution and added in each well. Goat anti-mouse alkaline phosphatase–conjugate antibodies at a final dilution of 1:2000 (Sigma Aldrich, Burlington, MA, USA) were used. Finally, a 100 mL/well of 3 mg/mL paranitrophenyl-phosphate disodium hexahydrate (Sigma Aldrich Burlington, MA, USA) in 1 M diethanolamine buffer pH 9.8 and plates were incubated at room temperature in the dark for 45 min. Absorbance was read at 405 nm using Varioskan™ LUX multimode (Thermo Fisher Scientific, Waltham, MA, USA) microplate reader.

### 2.17. Preparation of Viral Pseudotypes and Neutralization Assays

Lentiviral particles pseudotyped with SARS-CoV-2 spike were produced in 10 cm plates prepared the day before transfection with 3 million HEK293T cells in 10 mL complete DMEM, supplemented with 10% FBS. Simian Immunodeficiency virus (SIV)-based vectors were produced by transfecting cells using the Calcium Phosphate method with 17.5 μg of env-defective SIV-Mac239-GFP construct with GFP expressed in place of Nef and 2.5 μg of PCDNA3.1 encoding the WT SARS-CoV-2 spike (reference sequence Wuhan-Hu-1, accession number YP_009724390) engineered to truncate the C-terminal 19 amino acids. Pseudotyped vector supernatants were harvested 48 h post-transfection and filtered through a 0.45 μm filter before. Neutralization titres were tested on Huh-7 cells overexpressing ACE2. Target cells were seeded onto a 96-well tissue culture one day before neutralization. The vector inoculum was adjusted to produce no more than 10% transduction of the monolayer to ensure a linear working range of the assay.

Sera dilutions were added to pseudotyped virus particles, incubated at room temperature for 30 min and added to cells. After 48 h, transduction was assessed by counting the GFP-expressing cells using the fluorescent plate reader Ensight (Perkin Elmer, Waltham, MA, USA). Each serum dilution was assessed in triplicate. Neutralization was measured by calculating the residual transduction activity of the pseudovirus considering the untreated sample as 100%. Fitted sigmoidal curves and IC50 were obtained using Prism (Graphpad) with the least-squares variable slope method and using the dose-normalized response protocol.

### 2.18. Statistical Analyses and Software

Detailed information concerning the statistical methods used is provided in the figure legends. Flow data were collected using FlowJo Version 10.5.3 (Treestar). Statistical analyses were performed with GraphPad Prism software version 8 (GraphPad). *n* represents individual mice analysed per experiments. Error bars indicate the standard error of the mean (SEM).

## 3. Results

### 3.1. Design and Construction of the OMV-Based SARS-CoV-2 Vaccine

**Figure 1:**
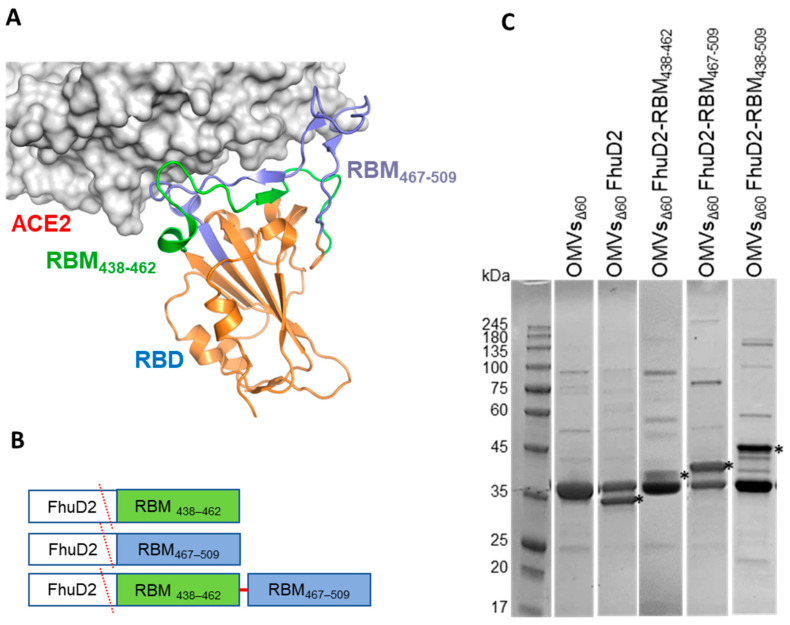
Construction and production of OMVs carrying SARS-CoV-2 RBM antigens. (**A**) Topology of the interaction between SARS-CoV-2 RBD and ACE2 with the indication of the two RBM polypeptides tested in this study. (**B**) Schematic representation of the pET21-based constructs expressed in BL21(DE3)*Δ60* to decorate OMVs. *Staphylococcus aureus* Ferric hydroxamate receptor 2 (FhuD2) was fused to one copy of RBM_438–462_, RBM_467–509_ and RBM_438–509_ SARS-CoV-2 epitopes. (**C**) SDS-PAGE of purified OMVs derived from BL21(DE3)*Δ60* cultures expressing pET21-FhuD2-RBM_438–462_, pET21-FhuD2-RBM_467–509_ and pET21-FhuD2-RBM_438–509_ plasmids or the control pET21-FhuD2 or empty pET21 plasmids. Stars indicate the predicted migration of the fusion proteins.

As shown by the 3D structure of the SARS-CoV-2 RBD in complex with ACE2, the RBM of the spike protein is organized in two regions spanning β5 and β6 strands, which include a majority of the spike residues contacting ACE2 (Figure 1A). Accordingly, patient-derived monoclonal antibodies (mAbs) binding these RBD chains potently inhibit SARS-CoV-2 entry in vitro and protect different animals from viral challenge [24,25,26,27,28]. These observations confirm the crucial role of RBM in engaging the receptor and demonstrate its immunogenicity in vivo. A number of mAbs targeting the RBM are currently in clinical use or in advanced clinical development [29].

In the attempt of producing a vaccine eliciting neutralizing immunity against SARS-CoV-2, we generated OMVs decorated with polypeptides derived from the RBM of the ancestral Wuhan-Hu-1 strain. The RBM polypeptides were fused to FhuD2, a *S. aureus* lipoprotein that can efficiently deliver heterologous polypeptides to the outer membrane and vesicular compartment in *E. coli* [8].

To this end, the nucleotide sequence encoding the strands β5 (RBM_438–462_) and β6 (RBM_467–509_) or a combination of the two (RBM_438–509_) were fused to the 3′ end of the sequence encoding FhuD2 in plasmid pET-FhuD2 [9] (Figure 1B). The resulting plasmids were used to transform the *E. coli BL21(DE3)Δ60* strain, a hyper-vesiculating *E. coli* BL21(DE3) derivative recently created in our laboratories [8], thus creating the three recombinant strains *E. coliΔ60(*pET-FhuD2-RBM_438–462_), *E. coli Δ60(*pET-FhuD2-RBM_467–509_) and *E. coliΔ60(*pET-FhuD2-RBM_438–509_). The OMVs from each recombinant strain, secreted into culture supernatants, were concentrated and purified. SDS-PAGE analysis revealed protein species with molecular weights corresponding to the designed fusion proteins (Figure 1C). Notably, the yields of the chimeric proteins were comparable with those of the free FhuD2 carrier. Therefore, the addition of the SARS-CoV-2 RBM polypeptides was well tolerated and compatible with the efficient transport into the bacterial vesicles.

### 3.2. Immunogenicity of FhuD2-RBM-OMVs

**Figure 2:**
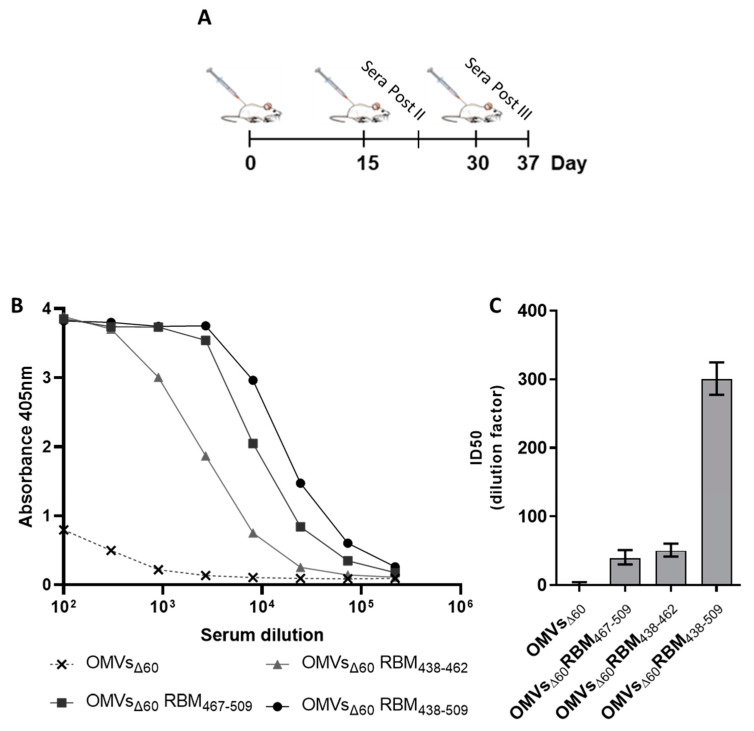
Mice immunized with OMVs decorated with SARS-CoV-2 RBM antigens produce neutralizing antibodies targeting the RBD. (**A**) Diagram of the experimental setup of the immunization experiments. CD1 mice received three intraperitoneal immunizations on days 0, 14 and 28 with 10 μg of OMVs together with 2 mg/mL aluminium hydroxide. Sera were collected 7 days after the last immunization. As control, mice were immunized with non-engineered OMVs. (**B**) Antibody titres in sera pooled from each group of 5 mice, collected 7 days after the third immunization, measured using ELISA, using plates coated with the SARS-CoV-2 RBD. (**C**) Neutralization activity in pooled sera, measured with SIV-based lentiviral vectors pseudotyped with SARS-CoV-2 spike from the ancestral isolate. The respective TCID50 values calculated with GraphPad are reported. The average values and the standard deviations from three determinations are plotted.

Having proven efficient production of FhuD2-RBM OMVs, we tested their ability to induce the production of antibodies capable of recognizing the RBM in the context of the SARS-CoV-2 Spike protein. To this aim, five CD1 mice were immunized with each construct three times at two-week intervals using 10 μg of each engineered OMV preparation combined with 2 mg/mL of Alum administered intraperitoneally (Figure 2A). Blood samples were collected seven days after the second and the third dose. RBD-specific IgGs in the pooled samples were detected by ELISA using recombinant Wuhan-Hu-1 RBD on solid support. After three immunizations, the sera from animals immunized with the three different FhuD2-RBM-OMVs contained significant amounts of IgGs recognizing the RBD (Figure 2B). Notably, the highest titres were detected in sera from mice immunized with the fusions carrying the β5-β6 polypeptide.

After three immunizations, the anti-RBD antibody titres were approximately five-fold higher than those achieved after two doses (Figure S1A). OMVs are known to induce Th1-skewed immune responses [12,30,31]. Given the importance of the Th1 profile for SARS-CoV-2 immunity [32], we analysed the type of immune response elicited by Alum-formulated RBM_438–509_-OMVs, the most immunogenic construct, by analysing the IgG isotype profile. As shown in Figure S1C, IgG2a represented a large proportion of the total IgGs. As a reference, we determined the total anti-RBD IgGs, IgG2a and IgG1 induced by the adenovirus-based ChAdOx1 nCoV-19 commercial vaccine, known to induce a potent Th1-skewed immune response [33]. As shown in Figure S1D, the IgG2a/IgG1 ratios of the OMV-based vaccine and ChAdOx1 nCoV-19 vaccine were comparable.

Having demonstrated the presence of anti-Spike antibodies with OMVs incorporating any of the three RBM peptides, we next asked whether such antibodies could also neutralize virus infection in vitro. To this end, neutralization was assessed through a pseudovirus assay using a GFP-encoding lentiviral vector based on SIV and pseudotyped with the SARS-CoV-2 Spike protein. Sera derived from each group of mice immunized with the engineered OMVs were pooled, serially diluted and combined with the pseudotyped vectors before inoculation on Huh-7-hACE2 target cells.

The data shown in Figure 2C and Figure S2 demonstrate that after three immunizations, the sera collected from mice immunized with any of the three RBM OMVs neutralized SARS-CoV-2 spike pseudotyped vectors. The OMVs that incorporated the single strands (RBM_438–462_ and RBM_467–509_) elicited neutralizing antibodies with similar potency (ID50 of 1:50 and 1:39, respectively). However, OMVs with the combination of the two beta strands (RBM_438–509_) induced a more powerful neutralization (ID50 of 1:301). These data indicated that both chains contributed and synergized to elicit potent neutralizing antibodies. Interestingly, the neutralizing activity elicited by the RBM_438–509_-OMVs in mice was of similar magnitude compared with the neutralization induced by the natural infection observed in convalescent patients which was measured using the same pseudovirus neutralization assay [34]. Two immunizations with RBM_438–509_-OMVs also produced good neutralization titres (Figure S1B). Therefore, only RBM_438–509_-OMVs were used in the subsequent experiments, henceforth called RBM_ancestral_-OMVs.

### 3.3. Protective Activity of RBM_ancestral_-OMVs in hACE2-Transgenic Mice

**Figure 3:**
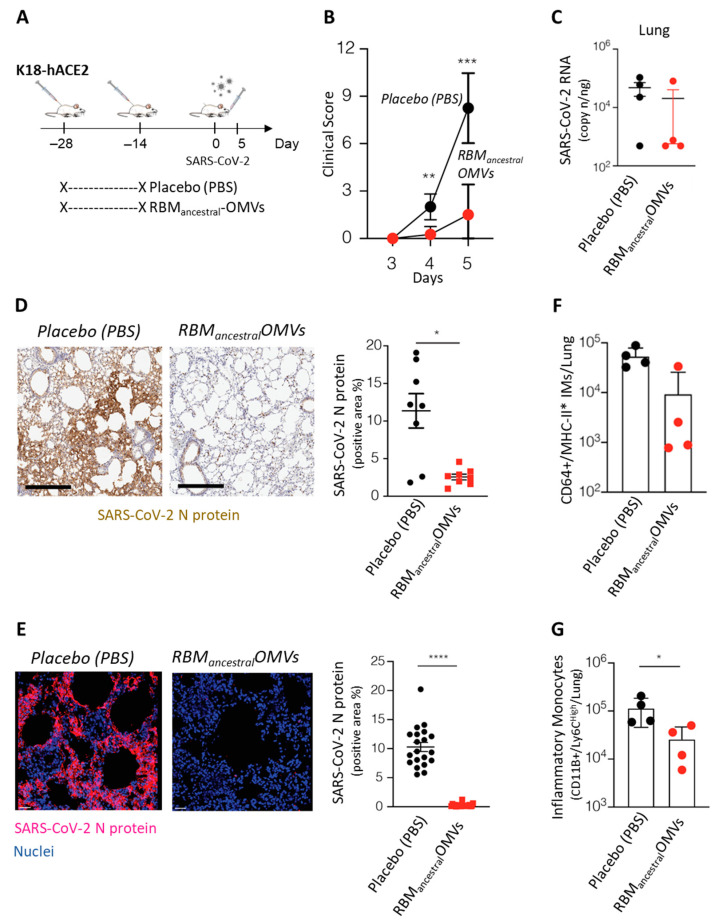
Immunization with RBM_ancestral_- OMVs protects against SARS-CoV-2 challenge in hACE2-transgenic mice. (**A**) Schematic representation of the experimental setup. K18-hACE2 mice (C57BL/6 background) received two intraperitoneal immunizations (at days −28 and −14) of 10 μg of OMVs vaccine (n = 4) or Placebo (PBS) (n = 4) prior to intranasal infection with 1 × 10^5^ TCID50 of SARS-CoV-2. Lung were collected and analysed five days after SARS-CoV-2 infection. (**B**) Mice were observed daily for clinical symptoms to assess severity of disease based on respiration, coat condition, posture, social behaviour, and palpebral aperture. (**C**) SARS-CoV-2 RNA in the lung was quantified by quantitative PCR with reverse transcription (RT–qPCR) 5 days after infection. (**D**) Representative immunohistochemical micrographs of lung sections 5 days post SARS-CoV-2 infection. N-SARS-CoV-2 expression is shown in brown. Scale bars, 300 μm. Right panel, quantification of N-SARS-CoV-2 signal, each dot represents a mouse. (**E**) Representative confocal immunofluorescence micrographs of lung sections from PBS-treated mice (left) or RBM_ancestral_-OMVs immunized mice (right) 5 days post SARS-CoV-2 infection. N-SARS-CoV-2-positive cells are depicted in purple and nuclei in blue. Scale bar represents 30 μm. Right panel, quantification of N-SARS-CoV-2 signal, each dot represents a different stack. (**F**) Absolute numbers of CD11b+/Ly6Chigh inflammatory monocytes in the lung of the indicated mice 5 days after SARS-CoV-2 infection. (**G**) Absolute numbers of CD64+/MHC-II+ inflammatory monocytes in the lung of the indicated mice. * *p* value < 0.05, ** *p* value < 0.01, *** *p* value < 0.001, **** *p* value < 0.0001.

Having established that the RBM_ancestral_-OMVs are effective at eliciting neutralizing immunity in mice, we explored its degree of protection against SARS-CoV-2 in vivo using K18-hACE2-transgenic mice [35]. A double-shot immunization regime was proven effective at the beginning of the COVID-19 pandemic, and likewise, two immunizations with RBM_ancestral_-OMVs were sufficient to elicit neutralizing antibodies (Figure S1B). Therefore, we first tested our OMV-based vaccine using a two-dose vaccination schedule. Groups of four mice each received two immunizations (at days −28 and at day −14) of either 10 μg of RBM_ancestral_-OMVs (*n* = 4) or Placebo (PBS) (*n* = 4) and fourteen days after the boost immunization, all mice were infected intranasally with 1 × 10^5^ TCID_50_ of SARS-CoV-2 (hCoV-19/Italy/LOM-UniSR-1/2020; GISAID Accession ID: EPI_ISL_413489) (Figure 3A). As expected [36], beginning 4 days post infection (p.i.) PBS-treated K18-hACE2-transgenic mice infected with SARS-CoV-2 developed a severe disease, as assessed by monitoring respiration, coat condition, posture, social behaviour, and palpebral aperture [37] (Figure 3B). By contrast, three out of four K18-hACE2-transgenic mice immunized with the RBM_ancestral-_OMVs vaccine were fully protected, as judged by the clinical score (Figure 3B). Moreover, viral RNA was almost undetectable in lungs of these mice (Figure 3C), indicating the ability of the vaccine to prevent virus replication in the respiratory tract. Mirroring the relative abundance of viral RNA, the N protein was also readily detected by both immunohistochemistry and immunofluorescence microscopy in lungs of PBS-treated mice, while it was mostly absent in mice immunized with the RBM_ancestral_-OMVs (Figure 3D,E).

The effective protection by the vaccine should also reflect the absence or attenuation of immune activation in response to the viral challenge. To assess the degree of inflammation triggered by the viral infection, we measured inflammatory monocytes (CD11b + Ly6C high and CD64+) in the lungs of infected animals. As shown in Figure 3F,G, after viral challenge, significantly less inflammatory monocytes were recovered from the lungs of vaccinated mice.

**Figure 4:**
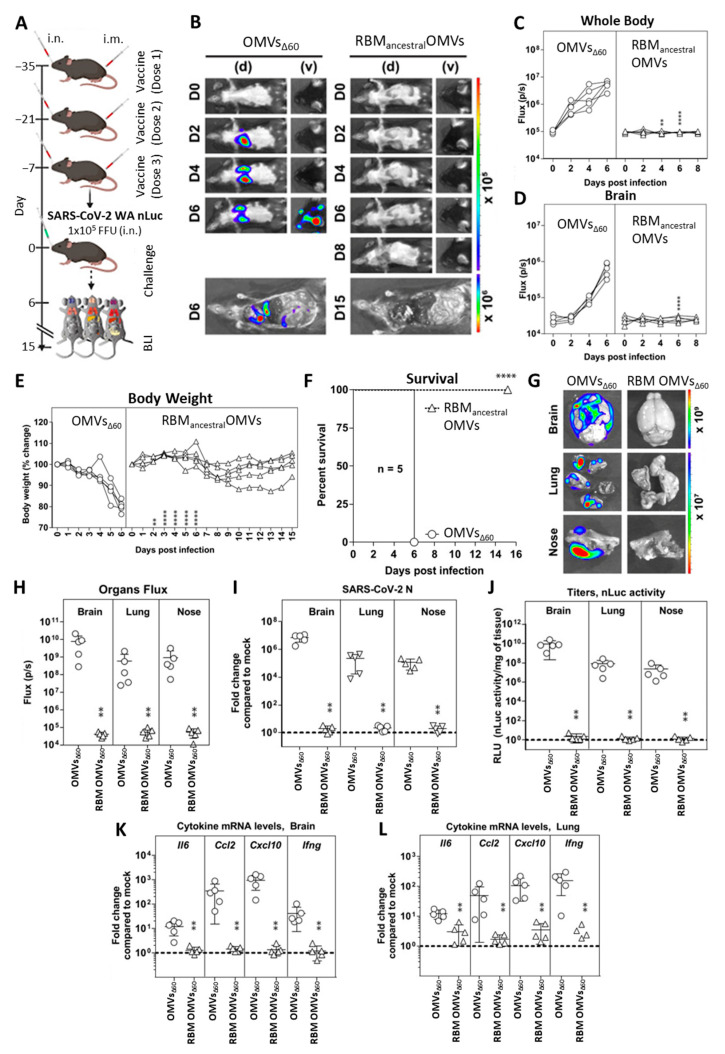
Immunization with RBM_ancestral_-decorated OMVs protects K18-hACE2 mice against lethal challenge with homologous SARS-CoV-2 ancestral strain. (**A**) Experimental design to evaluate the immunization efficacy of OMVs decorated with RBM_ancestral_-OMVs in K18-hACE2 mice challenged with SARS-CoV-2-WA-nLuc (i.n., 1 × 10^5^ FFU). Mice immunized with empty OMVs served as controls. Virus infection in mice was followed by non-invasive bioluminescence imaging (BLI) every 2 days from the start of infection. (**B**) Representative BLI images of SARS-CoV-2-WA-nLuc-infected mice in ventral (v) and dorsal (d) positions. Scale bars denote radiance (photons/sec/cm^2^/steradian). (**C**,**D**) Temporal quantification of nLuc signal as flux (photons/sec) computed non-invasively. (**E**) Temporal changes in mouse body weight with initial body weight set to 100. (**F**) Kaplan–Meier survival curves of mice (n = 5 per group) statistically compared by log-rank (Mantel–Cox) test for experiment as in (**A**). (**G**,**H**) Ex vivo imaging of indicated organs and quantification of nLuc signal as flux (photons/sec) after necropsy for an experiment shown in (**A**). (**I**) Fold change in SARS-CoV-2 nucleocapsid (N) mRNA expression in brain, lung and nose. (**J**) Viral loads (nLuc activity/mg) in indicated tissues measured after necropsy on Vero E6 cells as targets. (**K**,**L**) Fold change in indicated cytokine mRNA expression in brain and lung. The data were normalized to Gapdh mRNA expression in the same sample and that in non-infected mice after necropsy. Viral loads (**I**,**J**) and inflammatory cytokine profile (**K**,**L**) were determined at the time of death at 6 dpi or 15 dpi for surviving mice after necropsy. Each curve in (**C**–**E**) and each data point in (**H**–**L**) represents an individual mouse. Grouped data in (**C**–**E**) were analysed by 2-way ANOVA followed by Sidak’s multiple comparison tests. The data in (**C**–**F**,**H**–**L**) were analysed by *t*-test followed by non-parametric Mann–Whitney test ** *p* < 0.01; **** *p* < 0.0001; mean values ± SD are depicted.

To further confirm the protective efficacy of RBM_ancestral_-OMVs, we used a bioluminescence imaging (BLI) to monitor the impact of vaccination on replication and spread of reporter SARS-CoV-2 WA-nanoluc luciferase (nLuc) reporter virus in K18-hACE2 mice. K18-hACE2 mice are extremely sensitive to SARS-CoV-2-induced mortality where the virus spreads systemically to distal tissues such as brain, gastrointestinal tract, and testis in addition to nasal cavity and lung [20]. Hence, in this set of experiments, we tested a mixed immunization schedule according to which K18-hACE2 mice were immunized both intranasally and intramuscularly with either RBM_ancestral_-decorated OMVs or “empty” OMVs_60_ as control (Figure 4A). Based on a previous report [20], this regime should elicit both mucosal and peripheral immunity to facilitate protection of mice against SARS-CoV-2 challenge. Groups of five mice were immunized three times at two-week intervals with 10 µg of RBM_ancestral-_OMVs both intramuscularly and intranasally. We confirmed the presence of Spike-binding antibodies in the sera collected one day before challenge by ELISA (Figure S3). On day 35, mice were intranasally challenged with 1 × 10^5^ FFU SARS-CoV-2_WA1_nLuc. Non-invasive longitudinal bioluminescence imaging (BLI), followed by quantification of nLuc signals in the whole body and brain, revealed that the virus replicated unabatedly and reached the brain by 4 days post infection (dpi) in mice immunized with empty OMVs (Figure 4B–D).

By contrast, nLuc signals were undetectable in RBM_ancestral_-OMVs-immunized mice, suggesting effective inhibition of SARS-CoV-2 replication (Figure 4B–D). In accordance with the BLI, mice immunized with empty OMVs experienced gradual weight loss and succumbed to infection at 6 dpi, whereas those immunized with RBM_ancestral_-OMV experienced only mild weight loss (9–14 days) before recovery and demonstrated 100% survival (Figure 4E,F). nLuc signals measured after necropsy in isolated target organs (lung, brain, and nose) corresponded to viral loads (N mRNA expression, nLuc activity) and indicated significant inhibition of virus titres in Rac_kstraw_ OMV-immunized mice compared to empty OMV-immunized controls (Figure 4G–J). Analyses of inflammatory cytokine mRNA expression (*Il-6*, *Ccl2*, *Cxcl10*, and *Ifnγ*) in target organs also revealed a 10- to 1000-fold reduction in RBM_ancestral_-OMVs-vaccinated mice compared to empty OMVs (Figure 4K,L). Overall, these data confirm that vaccination with RBM_ancestral_-OMVs protected mice against lethal SARS-CoV-2 challenge by preventing both viral replication and the ensuing inflammatory cytokine response.

### 3.4. Engineering and Immunogenicity of OMVs Decorated with the RBM from Omicron BA.1 Variant

**Figure 5:**
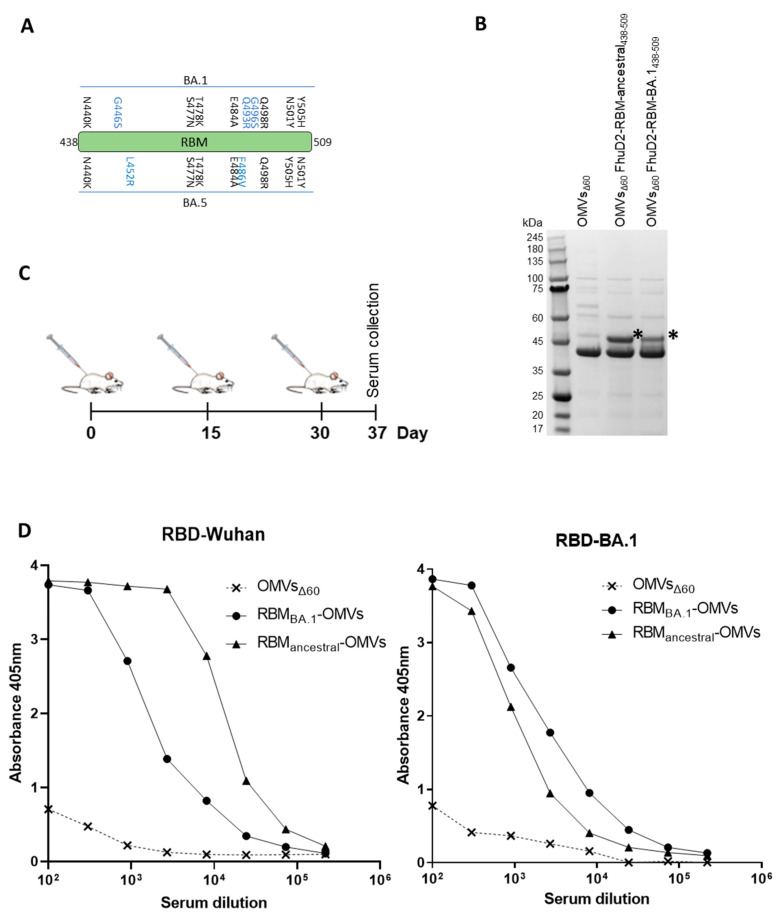
Production and immunogenicity of OMVs carrying SARS-CoV-2 RBM antigens derived from the omicron BA.1 isolate of SARS-CoV-2. (**A**) schematic of the residues found in micron BA.1 and BA.5 RBM which differ from to the reference ancestral strain sequence. Residues in blue are uniquely present in BA.1 and BA.5 strains. (**B**) SDS-PAGE of purified OMVs decorated with RBM from ancestral and omicron BA.1 strains generated by BL21(DE3)*Δ60* cultures expressing pET21-FhuD2-RBM_438–462_ and pACYC-FhuD2-RBM-BA.1_438–462_ plasmids, respectively, or the control empty pET21 plasmid. The asterisk (*) represents the band of expect size for both fusion constructs. (**C**) Diagram of the experimental setup of the immunization experiments. CD1 mice received three intraperitoneal immunizations on days 0, 15 and 30 with 10 μg of OMVs together with 2 mg/mL aluminium hydroxide. Sera were collected 7 days after the last immunization. As control, mice were immunized with non-engineered OMVs. (**D**) Antibody titres in sera pooled from each group of 5 mice, collected 7 days after the third immunization, measured using ELISA, using plates coated with the SARS-CoV-2 RBD from the ancestral strain (left) and omicron BA.1 (right).

To test the adaptability of the OMV platform, we engineered the vesicles with the combination of β5 and β6 strands (RBM) from the Omicron BA.1 variant, which carries 10 amino acid substitutions compared to the ancestral Wuhan-Hu-1 strain (Figure 5A). As shown in Figure 5B, RBM BA.1_438–509_ fused to FhuD2 was expressed in *E. coli* BL21(DE3)*Δ60* and efficiently compartmentalized in the OMVs. Next, we asked whether RBM_BA_._1_-OMVs could elicit antibodies capable of recognizing the corresponding purified RBD. Mice were immunized with the engineered OMVs following the time schedule described in Figure 5C and sera were collected seven days after the final injection. Antibody titres were assessed using ELISA coating the plates with either purified RBD_ancestral_ or purified RBD_BA_._1_. As shown in Figure 5D, RBM_BA_._1_-OMVs elicited IgGs, which recognized the homologous and the ancestral RBDs at similar titres. As a control, the pool of sera from mice immunized with RBM_ancestral_-OMVs was also tested against the two RBDs. In this case, the pool of sera recognized the homologous RBD at very high titres and the BA.1 RBD at titres similar to those measured in sera from mice immunized with RBM_BA_._1_-OMVs.

### 3.5. RBM_ancestral_-OMVs Elicit Cross-Neutralizing Antibodies

**Figure 6:**
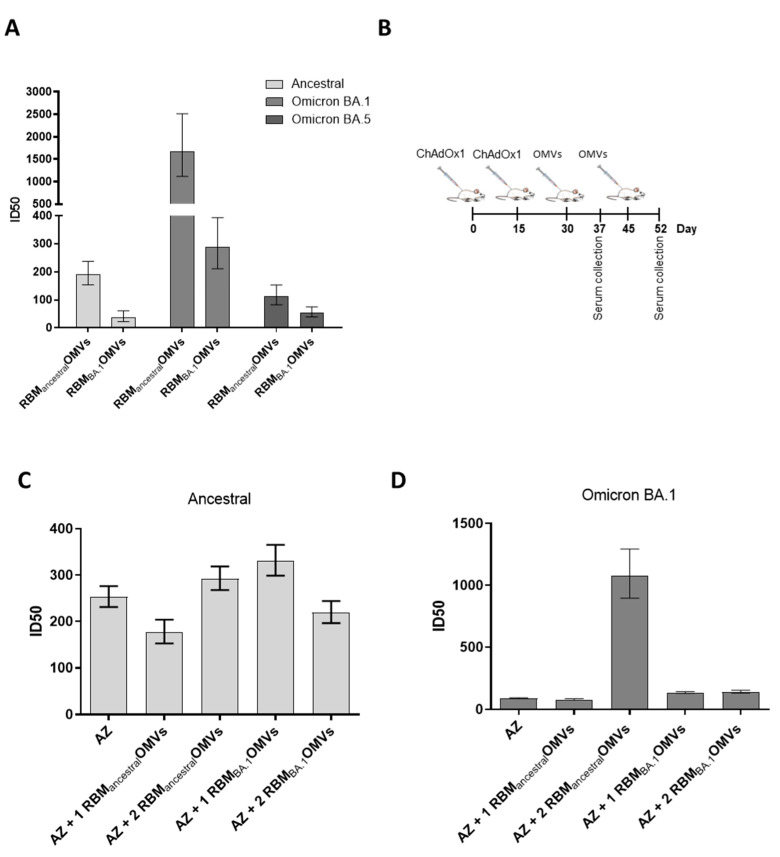
Ability of OMVs decorated with RBM antigens derived from the ancestral and omicron BA.1 isolates to boost immunity previously elicited by ChAdOx1 (AZ): (**A**) neutralization activity in pooled sera from animals immunized with OMVs decorated with RBM derived from the ancestral strain or the omicron BA.1 variant. Neutralization was measured with SIV-based lentiviral vectors pseudotyped with SARS-CoV-2 spike from the ancestral isolate, omicron BA.1 and BA.5 variants, plated on Huh-7 cells. Shown are ID50 values calculated from the curves shown in Figure S2 with GraphPad. Error bars represent the standard deviations from three determinations. (**B**) Diagram of the experimental setup of the immunization experiments. CD1 mice received two intramuscular immunizations with ChAdOx1 (AZ) on days 0 and 15 and two additional immunizations with OMVs decorated either with RBM from the ancestral or omicron BA.1 strains. Sera were collected prior to the second OMVs immunization or 7 days after the last immunization. As control, mice were immunized with non-engineered OMVs. (**C**,**D**) Neutralization activity in sera from animals immunized following the experimental setup in B, measured with lentiviral vectors pseudotyped with SARS-CoV-2 spike from the ancestral isolate and the omicron BA.1 variant, plated on Huh-7 cells. Shown are ID50 values calculated from the curves shown in Figure S3 with GraphPad. Error bars represent the standard deviations from three determinations.

Having observed substantial cross-reactivity using ELISA, we next tested whether the anti-RBD_BA_._1_ antibodies elicited by RBM_ancestral_-OMVs could also neutralize SARS-CoV-2 pseudotyped vectors expressing the omicron BA.1 and BA.5 variant spike proteins. This would be an unexpected result, considering that patients infected with the original ancestral strain and vaccinees that received ancestral Spike-based vaccines are poorly protected against the Omicron variants [38]. In addition, most monoclonal antibodies [39], which potently neutralize the ancestral strain by binding to the RBM region, are poorly effective against Omicron [40,41]. The sera collected from mice immunized with RBM_ancestral_-OMVs and RBM_BA_._1_-OMVs after three immunizations were tested for their in vitro neutralization capacity against the ancestral, the Omicron BA.1 and Omicron BA.5 pseudoviruses. As shown in Figure 6A and Figure S4, sera from RBM_BA_._1_-OMVs-immunized mice neutralized Omicron BA.1 pseudovirus with an ID 50 of 1:300. By contrast, the same sera were ineffective against ancestral and Omicron BA.5 (ID50 < 50), suggesting that the neutralizing antibodies elicited by omicron BA.1 RBM are highly variant-selective and therefore poorly cross-neutralizing. Such neutralization behaviour diverges from the binding activity of the same sera, observed with ELISA (Figure 5D), which indicates that antibodies from RBM_BA_._1_-OMVs-immunized mice bind the RBD derived from ancestral and Omicron BA.1 with equal efficiencies. Therefore, despite binding the RBD, antibodies elicited by RBM_BA_._1_ do not neutralize the ancestral pseudoviruses. In contrast, sera from animals that received RBM_ancestral_-OMVs displayed the ability to cross-neutralize all three SARS-CoV-2 variants tested, though with different efficiencies. While ancestral and omicron BA.5 pseudoviruses were neutralized with ID50 ranging from 1:100 to 1:200, omicron BA.1 pseudoviruses were neutralized with an average ID50 of 1:1.500 (Figure 6A). Therefore, RBM_ancestral_ displayed on OMVs has a greater ability than omicron RBM_BA_._1_ to elicit broadly neutralizing antibodies, against which omicron BA.1 pseudovirus was highly sensitive. Collectively, the results of these experiments indicate that RBM_ancestral_-OMVs has the potential to be an anti-COVID-19 vaccine with a broad protective activity.

Considering that a large proportion of the worldwide population has been either infected with SARS-CoV-2 or has been vaccinated with ancestral-based vaccines, we investigated the immune responses elicited by RBM-OMVs in animals that had previously received AstraZeneca ChAdOx1 nCoV-19 vaccine (AZ). The mice were vaccinated with RBM_ancestral_-OMVs twice after receiving two doses of AZ at two-week intervals. Sera were collected seven days after the AZ vaccination and seven days after the first and the second dose of the OMV-based vaccine (Figure 6B). All sera were tested for their capacity to neutralize both the ancestral strain and the Omicron BA.1 variant using the in vitro pseudovirus assay (Figure 6C,D and Figure S5). As expected, the pool of sera from AZ-immunized mice effectively neutralized the homologous pseudovirus (ID50 ranging between 1:200 and 1:320) while showing poor neutralization against the Omicron variant (ID50 < 100). A similar neutralization pattern was observed in sera from animals after the administration of a single dose of RBM_ancestral_-OMVs. By contrast, while not further increasing the neutralization capacity already elicited by AZ against ancestral pseudoviruses, two doses of RBM_ancestral_-OMVs dramatically increased the neutralization titres against BA.1 pseudoviruses (ID50 1:1.500), mirroring the effect of the immunization with RBM_ancestral_-OMVs alone (Figure 6A). These data suggest that the AZ vaccine elicits neutralizing antibodies which differ from those induced by RBM-OMVs immunization. In fact, AZ-immunized mice seemed to behave like “naive” animals with respect to OMV vaccination.

## 4. Discussion

We demonstrate for the first time that engineered *E. coli* OMVs can provide an ideal platform for the development of an effective SARS-CoV-2 vaccine. While properly folded eukaryotic glycoproteins can be typically expressed only in eukaryotic expression systems, the crucial portion of the Spike RBM, which directly contacts ACE2, can be efficiently incorporated into OMVs. An effective immunity is elicited in animals vaccinated with RBM_ancestral_-OMVs, with the production of neutralizing antibodies at levels sufficient to protect hACE2-transgenic mice from infection. This is a very exciting result, considering that OMVs are extremely easy to produce, upscale and distribute. The separation of the biomass from the culture supernatant and an ultrafiltration step to concentrate OMVs and eliminate contaminants is essentially all that is needed for vaccine production [16]. Crucially, the same process can be applied to any antigen and different vaccines and is amenable to large-scale production which can be easily transferred to different facilities worldwide. Moreover, the production yields make the vaccine costs particularly affordable. Under laboratory conditions, we reproducibly obtain more than 5000 OMV-based vaccine doses/litre of culture. Practically speaking, this means that with a 1.000 litre fermentation unit associated with a tangential flow ultrafiltration device it is possible to provide >1 million doses of vaccine/week at costs that are expected to be well below 1 USD/dose. Given the ease of production, upscaling, and product stability, OMV vaccines should favourably compete with RNA-based technologies.

The intranasal delivery of the RBM_ancestral_-OMV vaccine is likely to be effective in preventing infection in the upper respiratory tract and viral dissemination in different organs and tissues, including the brain. This can be at least partially inferred from the results of the two animal experiments described in this work. In the first set of experiments, we immunized hACE2-transgenic mice systemically and subsequently challenged them intranasally with the ancestral strain. Three out of four animals survived, and viruses were not detected, using both immunohistochemistry and PCR, in the lungs of the protected mice. However, the virus could be found in the brain tissue of both protected and mock-immunized mice. In the second set of experiments, the RBM_ancestral_-OMV vaccine was given both systemically and intranasally before challenge. Following this immunization schedule, all five immunized animals under this regimen were completely protected, and the virus could not be detected in the lung, brain and nose of the animals. Moreover, protection strongly correlated with the absence of inflammatory cytokines in the analysed tissues. Therefore, although we have not yet tested the intranasal immunization alone, the data suggest that mucosal delivery contributes substantially to overall protection. Such data would be in line with the data obtained with a mucosal vaccine based on the OMVs from *Neisseria meningitidis* combined with recombinant Spike protein [42].

Our work also showed that the RBM_ancestral_-OMVs vaccine elicits cross-neutralizing antibodies against different VOCs. The OMVs platform is flexible enough to be rapidly adapted to cope with new emerging variants. In addition to the RBM of the ancestral and BA.1 strain, we have successfully engineered our OMVs with the RBM from the P1 and B.1.617 isolates (not shown). All engineered OMVs elicited high levels of antibodies, which effectively neutralized the homologous pseudoviruses in vitro. Moreover, we found that the antibodies induced by the RBM_ancestral_-OMVs vaccine provided robust cross-protection against the most recent Omicron variants. This is remarkable, considering that the vaccines and the neutralizing monoclonal antibodies based on the ancestral Spike protein are known to be poorly effective against Omicron variants [40]. When expressed on OMVs, the RBM of the ancestral strain, but not the RBM of the BA.1 strain, presents otherwise poorly immunogenic epitopes, which induce antibodies against conserved structural regions of the RBD involved in the interaction with the ACE2 receptor. It would be of particular interest to isolate the B cells from the RBM_ancestral_-OMVs immunized mice and characterize these putative new classes of antibodies. The existence of a new family of neutralized antibodies would be of particular interest from a vaccination standpoint. Nowadays, most of the worldwide population has been either infected by SARS-CoV-2 or vaccinated. Therefore, the immunization with the RBM_ancestral_-OMVs vaccine should synergize with the natural immunity of the vaccinees, thus making the vaccination particularly effective.

While further experiments are needed to test the efficacy against a larger panel of SARS-CoV-2 variants, our data suggest that the RBM_ancestral_-OMVs vaccine has the potential to become a broadly protective vaccine that, thanks to its ease of manufacturing and low production costs, could be produced and distributed in low-income countries.

## Figures and Tables

**Table 1:** Oligonucleotides used for cloning SARS-CoV-2 RBM regions.

Name	Nucleotide Sequence
1-F	TAATTAAAGCTGCAAAAGATATTAGCACCGAAATTTATCAGGC
1-R	GATGGTGATGGTGATGTTAACGATACGGCTGATAACCCAC
2-F	TAATTAAAGCTGCAAAAAGCAATAACCTGGATAGCAAAG
2-R	GATGGTGATGGTGATGTTATTTCAGATTGCTCTTACGAAAC
F-1	CCGTTTGAACGTGATATTAGCACCGAAATTTATCAGG
PACYC-F	AGCCAGGATCCGAATTCGAGC
FhuD2-v-R	TTTTGCAGCTTTAATTAATTTTTC
pET-v-F	CATCACCATCACCATCACGATTACA
R-1	ACGTTCAAACGGTTTCAGATTGCTCTTACGAAAC

**Table 2:** Sequences of cloned SARS-CoV-2 RBM regions.

Name	Nucleotide Sequence
RBD_438–462_	AGCAATAACCTGGATAGCAAAGTTGGTGGCAACTATAACTATCTGTATCGCCTGTTTCGTAAGAGCAATCTGAAA
RBD_467–509_	GATATTAGCACCGAAATTTATCAGGCAGGTAGCACCCCGTGCAATGGTGTTGAAGGTTTTAATTGTTATTTTCCGCTGCAGAGCTATGGTTTTCAGCCGACAAATGGTGTGGGTTATCAGCCGTATCGT
RBD_438–509_	AGCAATAACCTGGATAGCAAAGTTGGTGGCAACTATAACTATCTGTATCGCCTGTTTCGTAAGAGCAATCTGAAACCGTTTGAACGTGATATTAGCACCGAAATTTATCAGGCAGGTAGCACCCCGTGCAATGGTGTTGAAGGTTTTAATTGTTATTTTCCGCTGCAGAGCTATGGTTTTCAGCCGACAAATGGTGTGGGTTATCAGCCGTATCGT
RBD-BA.1_438–509_	AGCAATAAACTGGATAGCAAAGTTAGCGGCAACTATAACTATCTGTATCGCCTGTTTCGTAAGAGCAATCTGAAACCGTTTGAACGTGATATTAGCACCGAAATTTATCAGGCAGGTAACAAACCGTGCAATGGTGTTGCGGGTTTTAATTGTTATTTTCCGCTGCGTAGCTATAGCTTTCGCCCGACATATGGTGTGGGTCATCAGCCGTATCGT

**Table 3:** Description of mouse clinical score.

Respiration	slight alteration	1
	moderate alteration	2
	marked alteration	3
Coat condition	slight piloerection	1
	moderate piloerection	2
	marked piloerection	3
Posture	hunched posture	1
Social behavior	reduced interaction with animals	1
	marked reduced interaction with animals	2
	lack of grooming	2
	passive behaviour + tremors	3
Palpebral aperture	open	0
	half closed	2
	sunken and half closed	3

## Data Availability

All data supporting the findings of this study are available within the paper and its Supplementary Materials.

## Supplementary Materials

The following supporting information can be downloaded at https://www.mdpi.com/article/10.3390/vaccines11101546/s1. Figure S1: Mice immunized with OMVs decorated with SARS-CoV-2 RBM antigens produce neutralizing Th1 antibodies targeting the RBD. Figure S2: Neutralization activity against Ancestral SARS-CoV-2 isolate of sera from mice immunized with OMVs decorated with SARS-CoV-2 RBM_ancestral_ antigens. Figure S3: hACE2-transgenic B6 mice immunized with OMVs decorated with SARS-CoV-2 RBM antigen produce neutralizing antibodies targeting the RBD. Figure S4: Cross-neutralizing activity of sera from mice immunized with OMVs decorated with SARS-CoV-2 RBM antigens. Figure S5: Ability of OMVs decorated with RBM antigens derived from the ancestral and omicron BA.1 isolates to boost immunity previously elicited by ChAdOx1(AZ); Table S1: Antibodies (Abs) used for Figure 4 flow cytometry data.
