# Supplementary material for: Immunogenicity and Pre-Clinical Efficacy of an OMV-Based SARS-CoV-2 Vaccine
Source: Vaccines (Basel). 2023 Sep 29;11(10):1546. doi: 10.3390/vaccines11101546 (PMC10610814; doi:10.3390/vaccines11101546)
Supplement: Supplementary file 1 [file vaccines-11-01546-s001.zip › vaccines-2569768-supplementary.pdf]

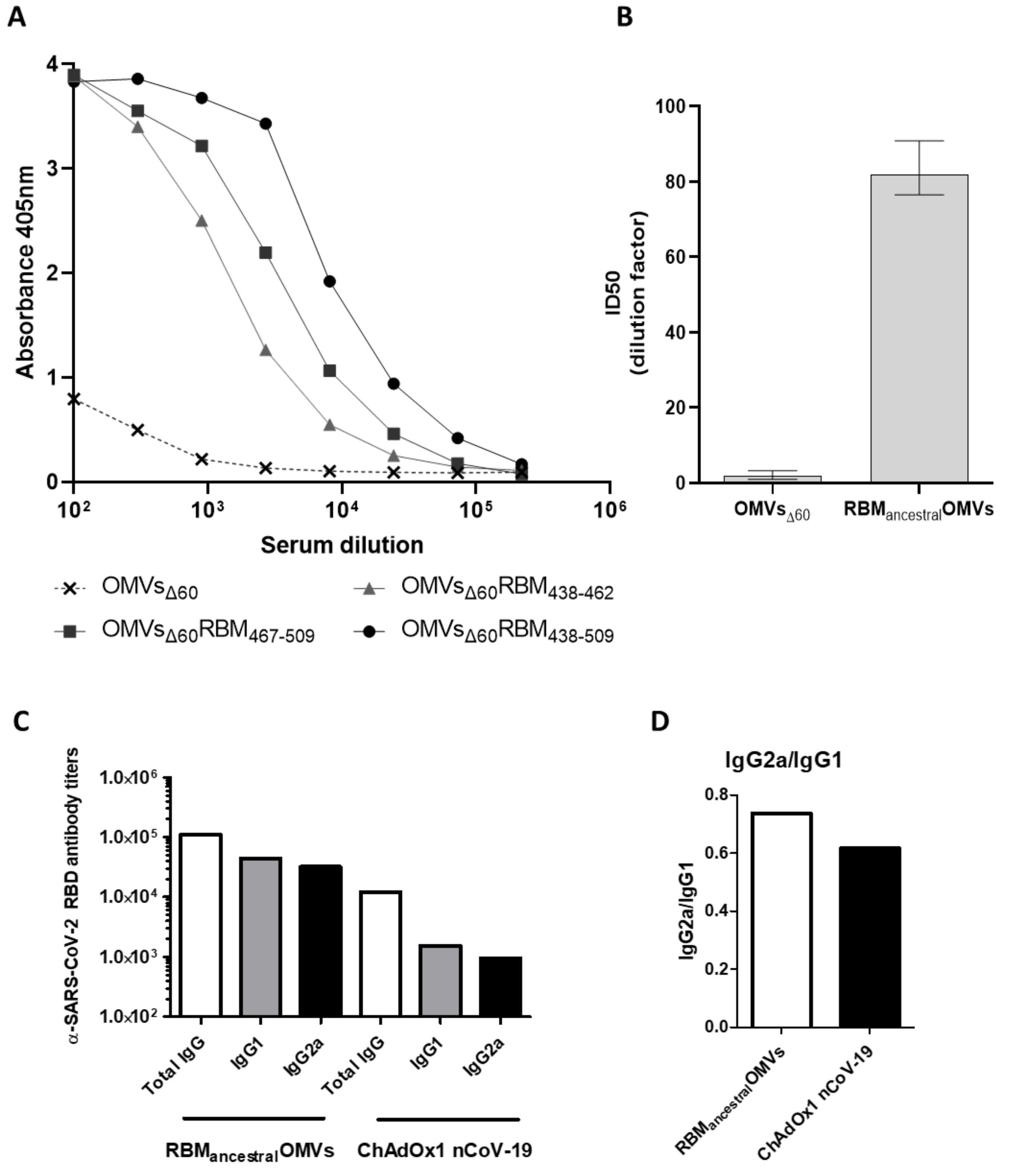

Figure S1. Mice immunized with OMVs decorated with SARS-CoV-2 RBM antigens produce neutralizing Th1 antibodies targeting the RBD. (**A**) Antibody titers in sera pooled from each group of 5 mice, collected seven days after the second immunization, measured by ELISA, using plates coated with the SARS-CoV-2 RBD. (**B**) Neutralization activity in pooled sera collected seven days after the second immunization, measured with lentiviral vectors pseudotyped with SARS-CoV-2 spike from the ancestral isolate. The respective TCID50 values calculated with GraphPad are reported. The average values and the standard deviations from three determinations are plotted. (**C**) Antibody titers and IgG isotypes in sera pooled from each group of 5 mice, collected seven days after the third or the second immunization for RBMancestral OMVs and ChAdOx1 nCoV-19 vaccine, respectively, measured by ELISA, using plates coated with the SARS-CoV-2 RBD. (**D**) IgG2a/IgG1 ration in sera of mice immunized with either for RBMancestral OMVs or ChAdOx1 nCoV-19 vaccine.

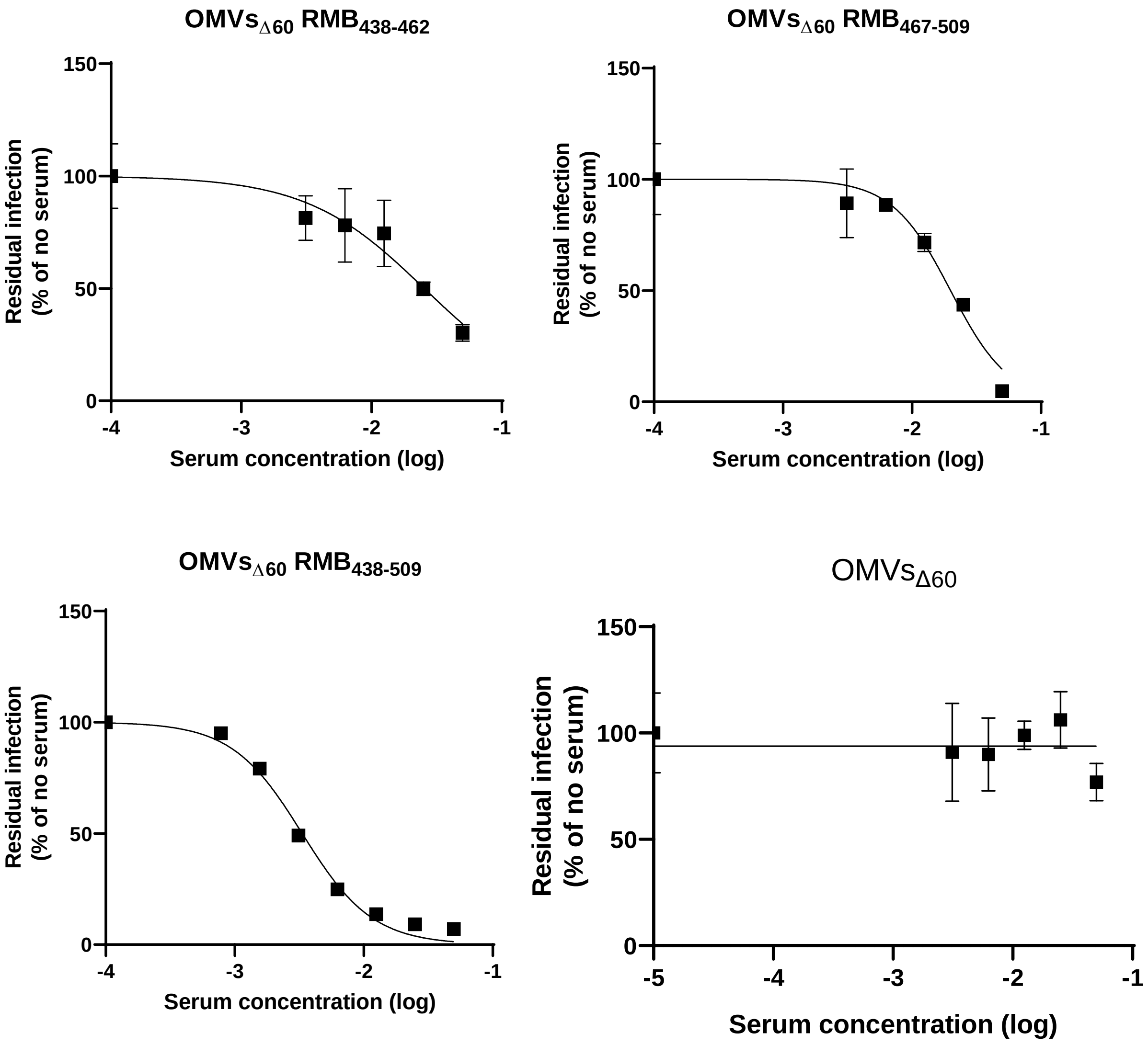

Figure S2. Neutralization activity against Ancestral SARS-CoV-2 isolate of sera from mice immunized with OMVs decorated with SARS-CoV-2 $RBM_{ancestral}$ antigens. Neutralization activity in pooled sera from mice immunized with SARS-CoV-2 RBMancestral antigens or empty OMVs (OMVsΔ60) as a control, measured with SIV-based lentiviral vectors pseudotyped with SARS-CoV-2 spike from the ancestral isolate, plated on Huh-7 cells. Residual infectivity after treatment with serially diluted sera, expressed as percentage of the untreated virus control.

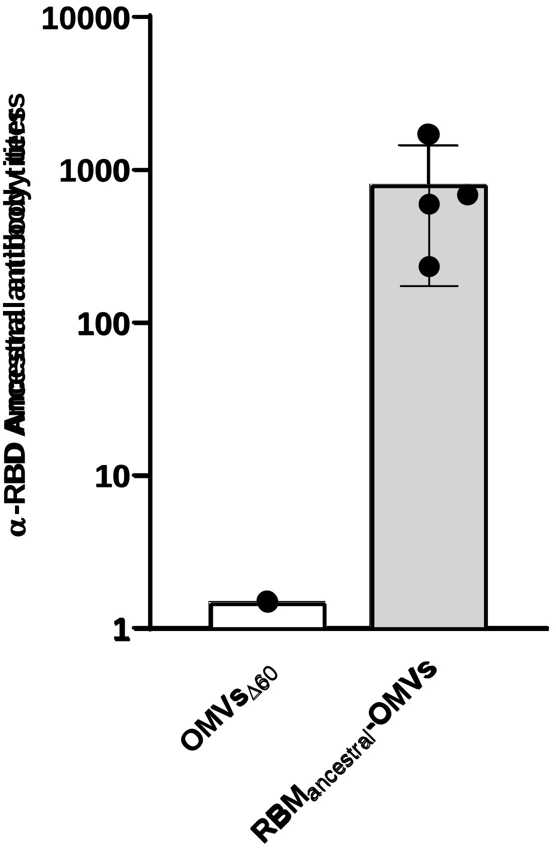

Figure S3. hACE2 transgenic B6 mice immunized with OMVs decorated with SARS-CoV-2 RBM antigen produce neutralizing antibodies targeting the RBD. Antibody titers in serum from single mouse, collected six days after the third s.c. and i.n. immunization, measured by ELISA, using plates coated with the SARS-CoV-2 RBD.

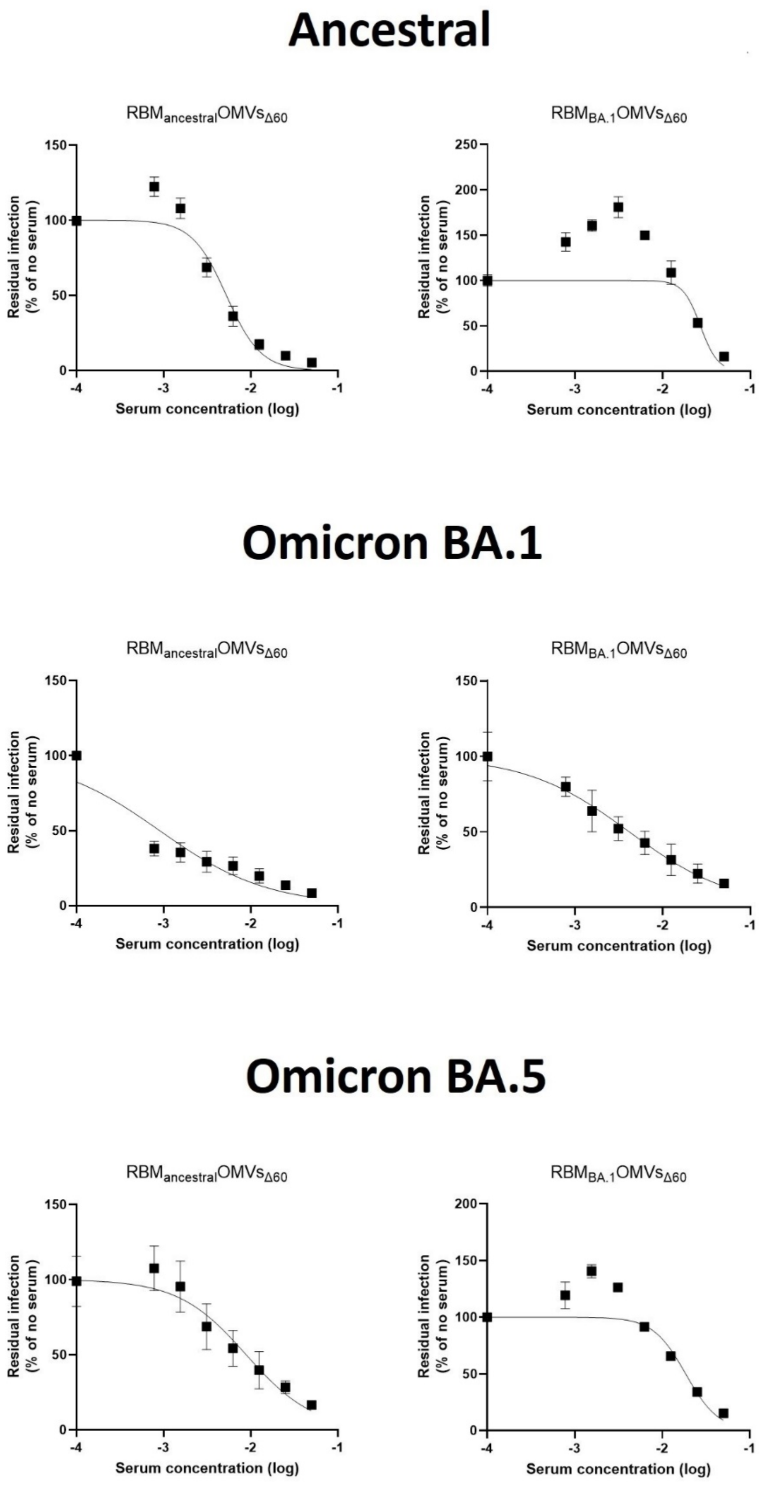

Figure S4. Cross-neutralizing activity of sera from mice immunized with OMVs decorated with SARS-CoV-2 RBM antigens. Neutralization activity in pooled sera derived from immunization with OMVs decorated with SARS-CoV-2 RBM antigens from the ancestral and omicron BA.1 strains, measured with lentiviral vectors pseudotyped with SARS-CoV-2 spike from the ancestral isolate and the omicron BA.1 and BA.5 isolates, plated on Huh-7 cells. Residual infectivity after treatment with serially diluted sera, expressed as percentage of the untreated virus control. Plotted are average values and standard deviations from triplicate determinations.

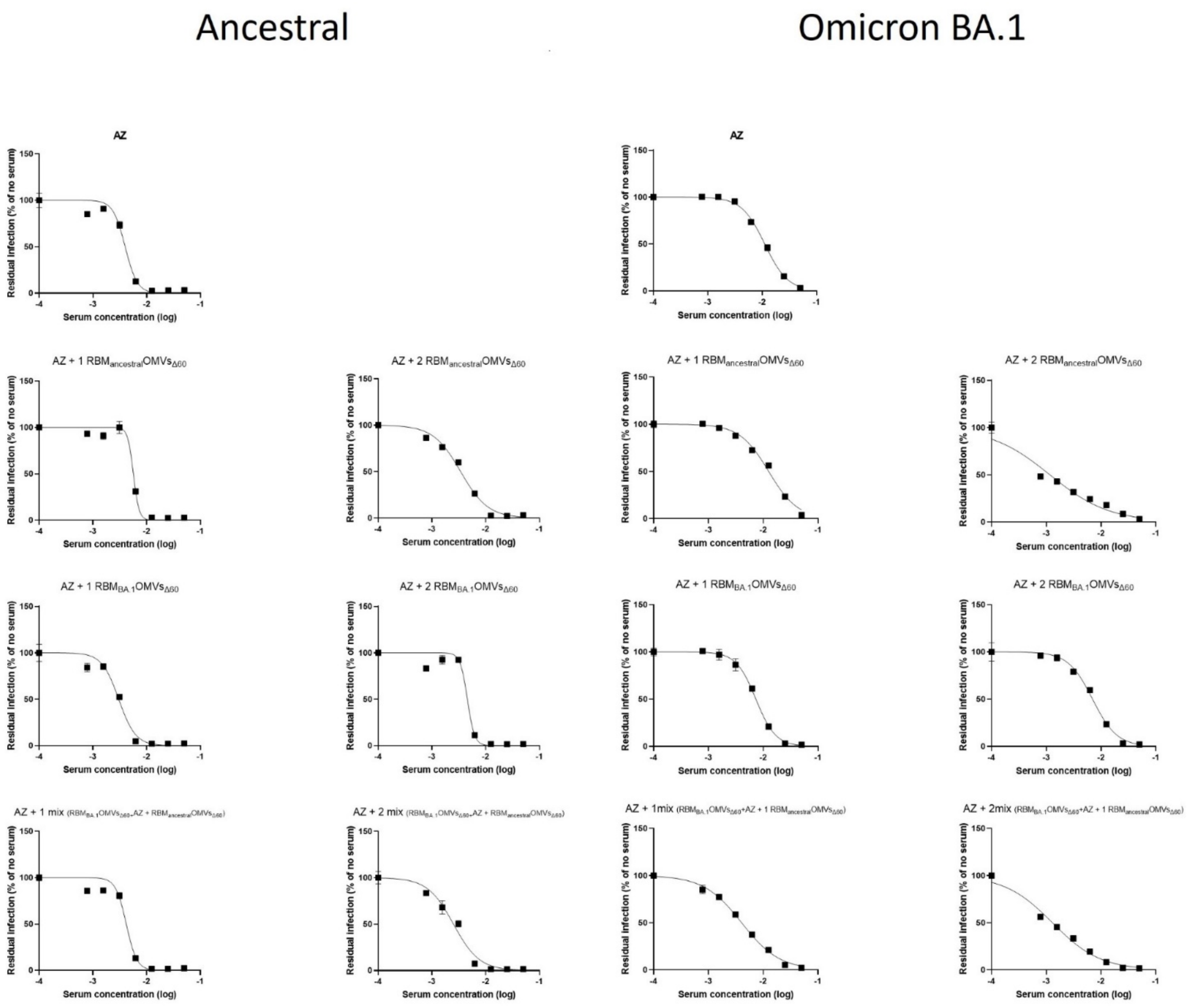

Figure S5. Ability of OMVs decorated with RBM antigens derived from the ancestral and omicron BA.1 isolates to boost immunity previously elicited by ChAdOx1(AZ). Neutralization activity in sera from animals immunized following the experimental setup in figure 6B, measured with lentiviral vectors pseudotyped with SARS-CoV-2 spike from the ancestral isolate and the omicron BA.1 variant, plated on Huh-7 cells. Plotted are average values and standard deviations from triplicate determinations.

Table S1 Antibodies (Abs) used for Figure 4 flow cytometry data

Name	Clone	Source and catalog number
CD103	2E7	Biolegend #121407
MHCII I-a/I-b	M5/114.15.2	Biolegend #107622
Ly-6C	HK1.4	Biolegend #128026
CD11b	M1/70	Thermo Fisher #48-0112-82
SIGLEC F	E50-2440	BD Bioscences #740388
CD64	X54-5/7.1	BD OptiBuild #740622
CD8	53-6.7	Biolegend #100759
CD11c	HL3	BD Bioscences #563735
CD4	RM4-5	BD Bioscences #740208
CD44	IM7	BD Bioscences #741227
CD69	H1.2F3	BD Bioscences #612793
F4/80	BM8	Biolegend #123110
Ly6g	1A5	BD Pharmingen #562700
CD45	30-F11	Biolegend #103113
